# RF tumor ablation with internally cooled electrodes and saline infusion: what is the optimal location of the saline infusion?

**DOI:** 10.1186/1475-925X-6-30

**Published:** 2007-07-16

**Authors:** Fernando Burdío, Enrique J Berjano, Ana Navarro, José M Burdío, Antonio Güemes, Luis Grande, Ramón Sousa, Jorge Subiró, Ana Gonzalez, Ignacio Cruz, Tomás Castiella, Eloy Tejero, Ricardo Lozano, Miguel A de Gregorio

**Affiliations:** 1 Department of Surgery, Hospital del Mar, Barcelona, Spain; 2 Center for Research and Innovation on Bioengineering, Valencia Polytechnic University, Valencia, Spain; 3 Department of Surgery A, Hospital Clínico Universitario Lozano Blesa, Zaragoza, Spain; 4 Department of Electric Engineering and Communications, University of Zaragoza, Spain; 5 Department of Urology, Hospital Clínico Universitario Lozano Blesa, Zaragoza, Spain; 6 Department of Animal Pathology and Surgery, Veterinary Faculty, University of Zaragoza, Spain; 7 Department of Pathology, Hospital Clínico Universitario Lozano Blesa, Zaragoza; 8 Department of Radiology, Hospital Clínico Universitario Lozano Blesa, Zaragoza, Spain

## Abstract

**Background:**

Radiofrequency ablation (RFA) of tumors by means of internally cooled electrodes (ICE) combined with interstitial infusion of saline may improve clinical results. To date, infusion has been conducted through outlets placed on the surface of the cooled electrode. However, the effect of infusion at a distance from the electrode surface is unknown. Our aim was to assess the effect of perfusion distance (PD) on the coagulation geometry and deposited power during RFA using ICE.

**Methods:**

Experiments were performed on excised bovine livers. Perfusion distance (PD) was defined as the shortest distance between the infusion outlet and the surface of the ICE. We considered three values of PD: 0, 2 and 4 mm. Two sets of experiments were considered: 1) 15 ablations of 10 minutes (n ≥ 4 for each PD), in order to evaluate the effect of PD on volume and diameters of coagulation; and 2) 20 additional ablations of 20 minutes. The effect of PD on deposited power and relative frequency of uncontrolled impedance rises (roll-off) was evaluated using the results from the two sets of experiments (n ≥ 7 for each PD). Comparisons between PD were performed by analysis of variance or Kruskal-Wallis test. Additionally, non-linear regression models were performed to elucidate the best PD in terms of coagulation volume and diameter, and the occurrence of uncontrolled impedance rises.

**Results:**

The best-fit least square functions were always obtained with quadratic curves where volume and diameters of coagulation were maximum for a PD of 2 mm. A thirty per cent increase in volume coagulation was observed for this PD value compared to other values (*P *< 0.05). Likewise, the short coagulation diameter was nearly twenty five per cent larger for a 2 mm PD than for 0 mm. Regarding deposited power, the best-fit least square function was obtained by a quadratic curve with a 2 mm PD peak. This matched well with the higher relative frequency of uncontrolled impedance rises for PD of 0 and 4 mm.

**Conclusion:**

Saline perfusion at around 2 mm from the electrode surface while using an ICE in RFA improves deposition of energy and enlarges coagulation volume.

## Background

Since the time when radiofrequency ablation (RFA) was described as a local therapy capable of destroying local liver malignancies, radiologists and surgeons have shown a growing interest in this technique, as it could provide a minimally invasive approach to treating many formerly untreatable patients [1,2]. Early work with RFA was limited by the small ablation volume that could be consistently achieved [3,4]. The rapid increase of temperature (above 100°C) at certain points of the tissue during RFA, leading to charring of the tissue and increase of impedance, was shown to be the main cause of the small coagulation volumes [5,6]. Since then many other strategies have been designed to improve energy deposition on tissue and further increase coagulation volume, mainly using either multitined and expandable, or internally cooled electrodes (ICE), with similar effectiveness [7,8].

**Figure 1:**
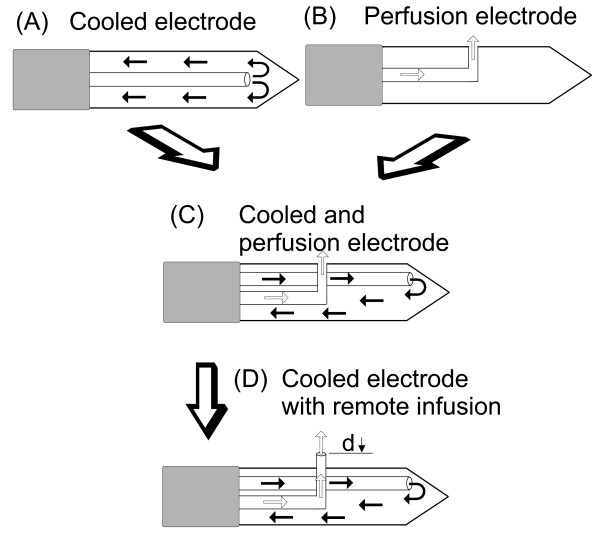
**Designs of RFA electrodes with internal cooling and/or saline perfusion into the tissue**. (A) Internally cooled electrode: water is circulated during ablation to cool tissue next to the applicator and prevent tissue charring. (B) Perfusion electrode: This allows the infusion of saline into tissue through one or more outlets on the electrode surface. (C) Hybrid applicator combining internal cooling and saline perfusion through outlets on the electrode surface. (D) Hybrid applicator combining internal cooling and saline perfusion through an outlet placed at a distance (d) from the electrode surface.

Water is circulated inside the ICE during ablation to cool tissue next to the applicator and prevent tissue charring (see Fig. 1A) [9]. They have proven utility and are largely employed in clinical practice, probably because they provide reliable coagulation zone geometry [10,11] while providing enough coagulation volume to treat medium or even large tumors (especially when cluster electrodes are used [12]).

On the other hand, perfusion electrodes provide saline infusion into tissue through one or more outlets on the electrode surface (see Fig. 1B). Even though they showed better efficiency in deposition of energy [13-15], they also gave distorted coagulation shapes [10] or an even higher rate of complications that have been linked to reflux of saline through the applicator path [16,17].

In order to obtain further increases of the coagulation zone in a reliable fashion, a combination of more than one system in the same applicator has recently been employed. These *hybrid *systems (according to the terminology of The International Working Group of Image-Guided Tumor Ablation [18,19]) usually try to combine the efficiency of perfusion electrodes with the reliability of either a multitined electrode [16,20] or ICE [21,22].

The benefit of a combination of saline perfusion into the tissue with ICE is an ongoing issue that is currently under evaluation (see Fig. 1C). Perfusion of saline into the tissue may both *improve conductivity *of the tissue and *transfer heat by convection in hot spots during RFA *[13], even though diffusion of saline into the liver tissue has not yet been characterized. So far, better performance has been demonstrated with this technique, both in deposition of energy and volume of coagulation, than the currently available technology either before [23] or during RFA [21,22,24-30]. Saline infusion into tissue has been performed either through an aperture in the electrode itself [21,22,24-28] or by other means [23,29,30] but, to our knowledge, the ideal location for saline infusion has not yet been defined.

Then again, Haemmerich et al [9] demonstrated that maximum temperatures were encountered about 2.5 mm away from the probe surface with conventional ICE. The same authors further demonstrated that beyond these hot spots, coagulation with ICE was mainly formed passively by thermal conduction. Keeping all these facts in mind, we hypothesized in our study that saline perfusion focused on the hottest areas of the tissue would be more efficient than either through the ICE itself (i.e., 0 mm distance) or at a distance from the hottest areas. Consequently, our aim was to assess the effect of perfusion distance (PD) on the efficacy of ablation. PD is taken as the shortest distance between the infusion outlet and the surface of the ICE (i.e. distance "d" in Fig. 1D). Efficacy was evaluated using a model based on excised bovine livers and by measuring coagulation volume, short coagulation diameter and energy deposition.

## Methods

The appropriate approval from the Local Ethical Committee was obtained before initiation of the experiments.

### RFA technique

**Figure 2:**
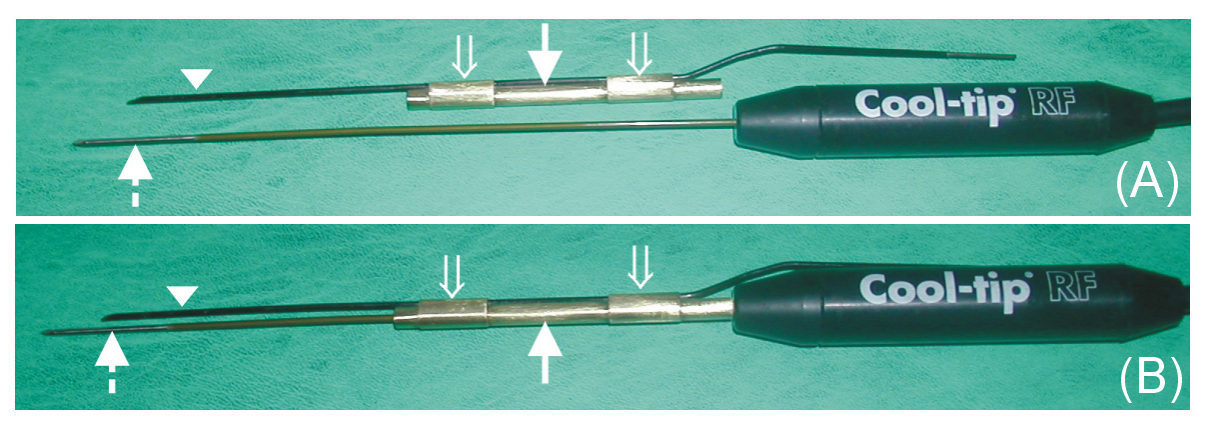
**Applicator used in the experiments to test the effect of perfusion distance**. An insulated perfusion needle (arrowheads) attached to a metallic outer sheath (solid arrows) by means of two fixation systems (open arrows) and a 17-gauge internally cooled electrode with a 3 cm exposed tip (broken arrows) (A). Once the metallic outer sheath is fitted to the internally cooled electrode, the perfusion needle remains parallel and midway from the exposed tip of the internally cooled electrode (B). The bottom photo shows the case of a perfusion distance of 2 mm.

Experiments were conducted with a 480 kHz generator model CC-1 (Radionics, Burlington, MA, USA) capable of producing currents up to 2000 mA (200 W). Simple internally cooled 17-gauge electrodes with 3 cm exposed tips (Cool-tip; Valleylab, Boulder, CO, USA) were employed. Since hypertonic infusion of saline was previously shown to increase conductivity and efficacy [13], we used NaCl 20% (at room temperature). This was infused at 100 mL/h by means of an Alaris IPX1 pump (Alaris Medical Systems, Basingstoke, UK), and injected through an independent 14-gauge needle assembled parallel by means of a metallic outer sheath and a fixation system attached to simple internally cooled 17-gauge electrodes with 3 cm of exposed tip (Fig. 2). It is important to point out that the exterior of the perfusion needle was insulated (i.e. plastic covered), and hence no electric field interference was expected between this needle and the exposed tip of the cooled electrode. A peristaltic pump (Watson Marlow, Wilmington, MA, USA) was used to deliver 0°C saline through the internally cooled electrodes at 10–25 mL/min, similarly to previous studies with internally cooled electrodes [13].

For all experiments, internal cooling of the electrode and perfusion of saline were begun at least 60 seconds before power deposition. The generator was set manually at 50, 100 W and maximum power at first, second and beyond third minute, respectively, similarly to Lee et al [21] and Kim et al [22]. The generator performed continuous monitoring of the impedance between the active part of the ICE and the grounding pad. Although the pulsed power algorithm has been demonstrated to improve coagulation volume and deposited power [31], it probably reduces the risk of charring the tissue. This algorithm was not employed in our study in order to better recognize charring and facilitate identification of impedance rises. Nonetheless, radiofrequency delivery was interrupted for 1 minute without interrupting either perfusion of saline or internal cooling if a spontaneous rise of impedance over 200 Ω was observed. The same power was then reapplied.

### Ex vivo experiments

**Figure 3:**
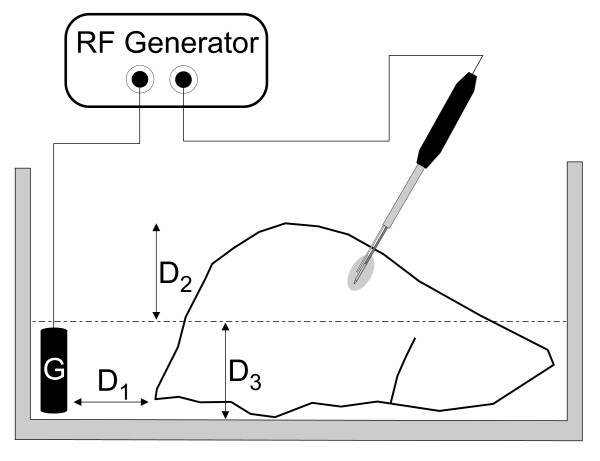
**Experimental setup of the ex vivo study**. Coagulation necrosis was induced at room temperature using bovine livers. The livers were partially immersed (D_2 _≈ D_3 _≈ 5–6 cm) in normal saline (NaCl 0,9%) at around 3 cm (D_1_) from a ≈ 200 cm^2 ^grounding pad, G.

Coagulation necrosis was induced at room temperature in bovine livers obtained from a slaughterhouse. The liver and the grounding pad (200 cm^2 ^total surface area) were immersed in normal saline (NaCl 0.9%) at a distance of around 3 cm from each other (see Fig. 3). We used an ICE with the perfusion needle assembled parallel to the outer metallic shaft and the fixation system (Fig. 1) in order to set three perfusion distance values: 0, 2 and 4 mm. Two sets of experiments were conducted:

i) The first set was performed to evaluate the effect of PD on coagulation volume and diameter. Fifteen ablations (n = 15) were performed for a period of 10 minutes each. To ensure experimental validity, at least four ablations were performed for each perfusion distance.

ii) The second set was planned to evaluate efficiency of energy deposition. Specifically, this was evaluated using the results from the first set of experiments (10-minute ablations) and 20 additional ablations of 20 minutes duration each. In the second set of experiments we specifically evaluated: a) spontaneous and uncontrolled rises of impedance or "roll-off" during RFA, defined as any increase of impedance over 200 Ω during the procedure [32], and b) mean deposited power throughout the ablation. In this set of experiments, at least seven ablations were performed for each value of perfusion distance.

### Assessment of coagulation necrosis and coagulation shape

**Figure 4:**
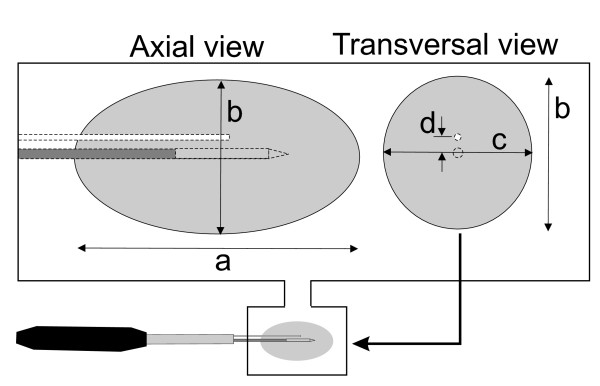
**Nomenclature employed to measure coagulation diameters**. After ablations, livers were sectioned along the longitudinal and transverse axes of the "white zone" of coagulation necrosis (gray in figure). Three diameters were measured by consensus of two observers: diameter along applicator track (a) and both perpendicular diameters (i.e., transverse diameters) to the applicator axis (b and c). The perfusion distance is d.

After RFA in each case, livers were sectioned along the longitudinal and transverse axes of the "white zone" of coagulation necrosis. As in Mulier et al [33], three diameters were measured for each RFA by consensus of two observers (see Fig. 4): diameter along applicator track (i.e. *a*-diameter) and both perpendicular diameters (i.e., transverse diameters) to the applicator axis (i.e. *b *and *c*-diameters). Volume was then calculated 1/6·π·*a·b·c*. These macroscopic findings ex vivo have been shown to correlate well with coagulation necrosis at histopathologic examination [12,31]. In all cases, the short diameter of the lesion was identified. The presence of charred tissue was also specifically assessed along the longitudinal coagulation axis, next to the applicator, where it is known to be most frequently located with conventional ICEs [9]. Charred tissue was defined, similarly to [34], as any dark tissue with or without cavitation, usually near the electrode path and surrounded by a pale or white zone of coagulation.

Coagulation shape was assessed by the sphericity ratio. This ratio was defined as the fraction of the largest and the average of the two remaining diameters in each lesion [10]. The closer this ratio is to 1, the more spherical the shape.

### Statistical analysis

Continuous data were compared by either analysis of variance (and Bonferroni posthoc analyses) or Kruskal-Wallis test when appropriate. Additionally, non linear fits (i.e. higher order regression models) and linear regression models were performed in order to determine the best locations for perfusion of saline in ex vivo studies. Hyperbolic functions were then fitted by using the classic method of least squares. The goodness of fit of a model was assessed by r^2 ^(coefficient of determination), which can be interpreted as the proportion of the total variability explained by the model. Differences with a value of α < 0.05 were considered to be statistically significant.

## Results

### Effect on coagulation volume, diameters and shape

**Figure 5:**
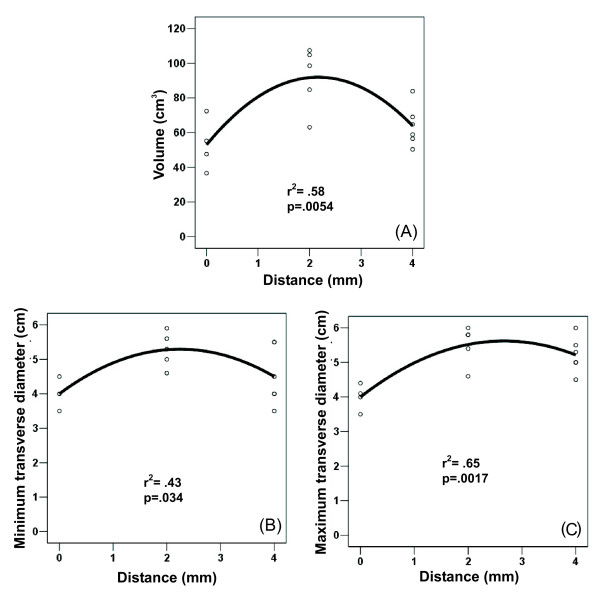
**Effect of perfusion distance on volume coagulation and coagulation diameters**. Three values of perfusion distance were tested: 0 mm (n = 4), 2 mm (n = 5) and 4 mm (n = 6). Scatter plot shows non-linear (quadratic) relationship between distance and coagulation volume (A), maximum transverse diameter of coagulation (B), and minimum transverse diameter (C). Goodness of fit of the models is individually assessed by r^2^. Note the improved effect at 2 mm distance.

Table 1 shows the results of the first set of experiments. In all cases, the short diameter of coagulation size was one of the two transverse diameters. The perfusion distance had a significant effect on volume, and also on minimum and maximum transverse diameters, as shown by the results of the analysis of variance. The best-fit least square functions were always obtained with quadratic curves for volume size (r^2 ^= 0.58; *P *= 0.0054) and both transverse diameters (Fig. 5). Volume size and both transverse diameters peaked their values for a perfusion distance of 2 mm.

**Table 1:** Effect of perfusion distance on coagulation volume, diameter and shape

**Perfusion distance (mm)**	**0 (n = 4)**	**2 (n = 5)**	**4 (n = 6)**	P*
**Volume size (cm^3^)**	53.0 ± 15.0	91.7 ± 18.2	63.9 ± 11.7	<0.05
**Axial diameter (cm)**	6.2 ± 1.0	6.0 ± 0.2	5.2 ± 0.5	NS
**Minimum transverse diameter (cm)**	4.0 ± 0.4	5.3 ± 0.5	4.5 ± 0.8	<0.05
**Maximum transverse diameter (cm)**	4.0 ± 0.4	5.5 ± 0.5	5.2 ± 0.5	<0.05
**Sphericity †**	1.5 ± 0.2	1.1 ± 0.1	1.1 ± 0.2	<0.01

The perfusion distance was defined as the shortest distance between the saline outlet and the surface of the internally cooled electrode

* Overall statistical significance. Statistically significant (*P *< 0.05). NS: no significant differences

† Fraction of the largest and the average of the two remaining diameters of the thermal lesion (see text)

The mean short diameter was greater for a perfusion distance of 2 mm than for the other perfusion distances (*P *< 0.05) (Table 1), but only the former comparison remained statistically significant in a posthoc analysis. Nevertheless, no significant differences were encountered in axial diameter for different perfusion distances, even though the greatest mean absolute values were obtained for a perfusion distance of 0 mm. Therefore, assessment of the sphericity ratio (Table 1) showed less spherical coagulation in the 0-mm group, compared to both 2-mm and 4-mm. Accordingly, a thirty per cent increase of mean volume size was demonstrated in the 2-mm group over the remaining groups (Table 1).

### Effect on energy deposition

**Figure 6:**
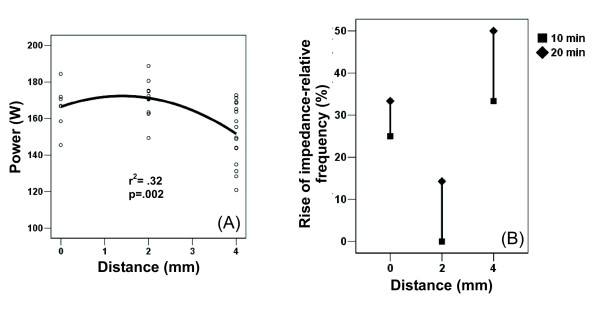
**Effect of perfusion distance on energy deposition into the tissue**. Three perfusion distances were tested: 0 mm (n = 7), 2 mm (n = 12) and 4 mm (n = 16). Scatter plot shows non-linear (quadratic) relationship between distance and deposited power (A). Goodness of fit of the model is assessed by r^2^. Note the improved effect at perfusion distance of 2 mm on deposition of power. Additionally, a drop-line chart (B) summarizes the observed relative frequency of an uncontrolled rise of impedance within two RFA durations (10 and 20 min) for the three values of perfusion distance. Note the lower rate of impedance rise during the procedure at a distance of 2 mm. In all cases, longer durations (20 minutes) were linked to a higher rate of impedance rise.

In the second set of experiments, the best-fit least square function was once more obtained with a quadratic curve for deposited power during the ablation (Fig. 6A). Deposited power during RFA thus peaked for a perfusion distance of 2 mm, reaching a mean value of 171.2 ± 9.8 W. This matched well with the higher relative frequency of experiments with any uncontrolled rise of impedance for perfusion distances of 0 and 4 mm (Fig. 6B). As expected, longer experiments (i.e. 20 minutes) were linked with higher frequency of uncontrolled impedance rise (Fig. 6B). The mean power found for perfusion distances of 0 and 4 mm were 166.3 ± 12.1 W and 151.6 ± 16.6 W, respectively.

**Figure 7:**
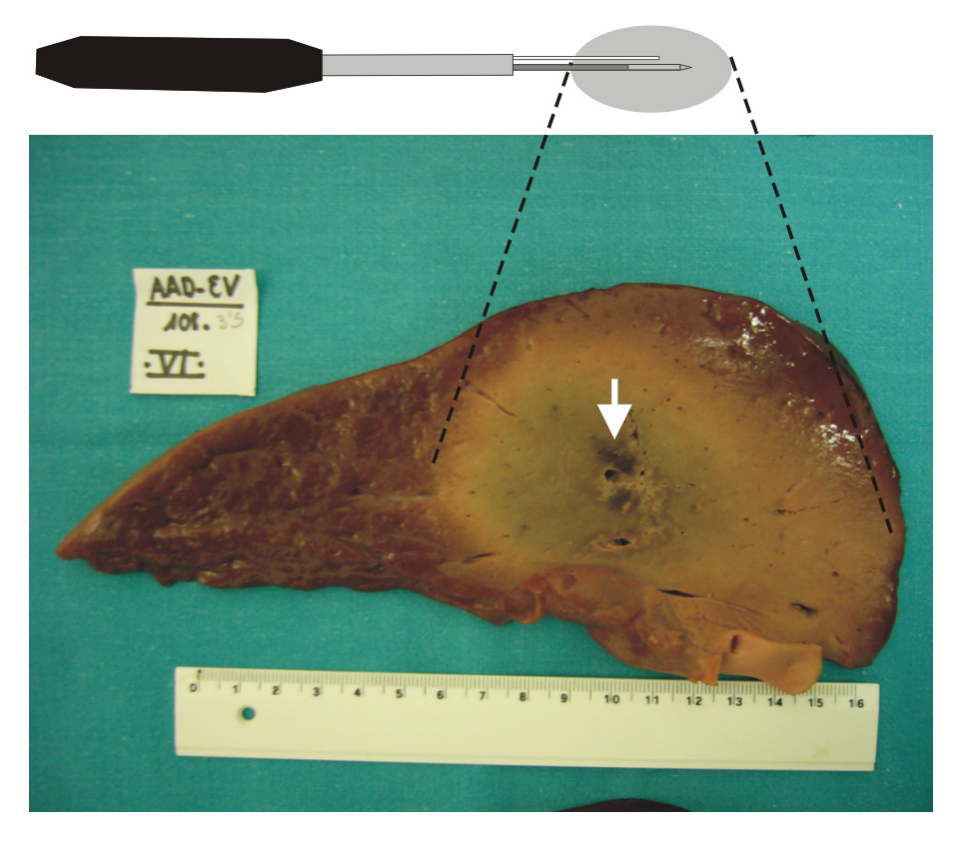
**Typical longitudinal section of the coagulation obtained for a perfusion distance of 4 mm**. Specimen in longitudinal section of the coagulation obtained by an internally cooled electrode combined with a perfusion distance of 4 mm. In this specimen a small irregular area of char tissue was observed (arrow) (scale in cm).

On macroscopic examination, several degrees of char tissue in irregular areas were observed in 12 (34.2%) of the experiments, usually near the electrode path (Fig. 7). It is noteworthy that in only 2 experiments (16.7%) in the 2-mm distance group was char tissue observed.

## Discussion

**Figure 8:**
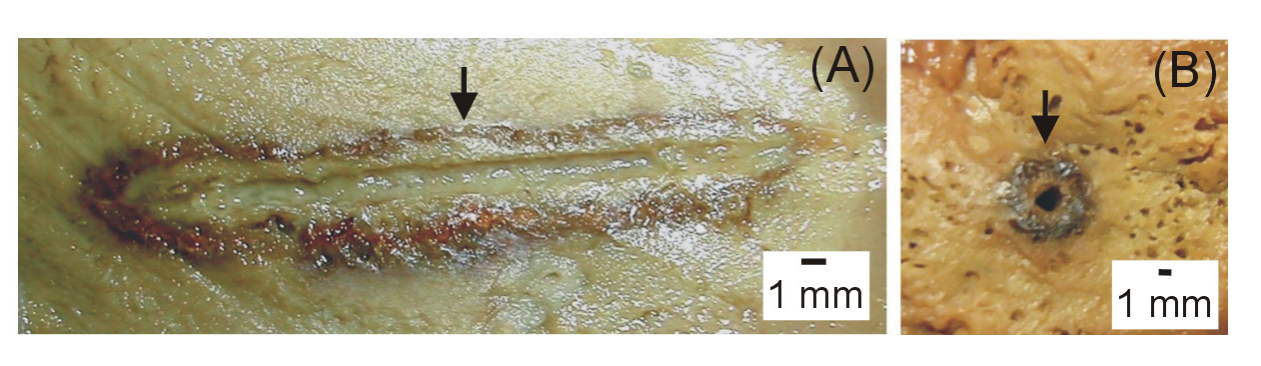
**Char ring created by a single internally cooled electrode with an effective area of 3 cm in length**. Longitudinal (A) and cross section (B) of the coagulation obtained by a single 3 cm exposed tip internally cooled electrode at 1800 mA (maximum current) for 10 minutes *without *using either saline perfusion into the tissue or an impedance control algorithm (unpublished personal experiments in bovine liver ex vivo). A ring of char tissue surrounding the electrode track is clearly shown (arrows).

In RFA of liver malignancies, the safe acquisition of a large ablation volume is of paramount importance for this technique to be accepted as a clinical routine [3]. In this setting, either internally cooled electrodes or multitined expandable electrodes have been shown to accomplish this goal better than perfusion electrodes [3,16]. In order to further increase coagulation zone size, a combination of more than one system has recently been employed in the same applicator. These *hybrid *electrodes usually combine perfusion of saline with either a multitined electrode [16,20] or an ICE [21,22]. Saline infusion is generally injected into the tissue either through an aperture at its active tip [21,22,24-28] or directly into the tissue itself [23,29,30]. Concerning RFA with ICE, Haemmerich et al [9] demonstrated that maximum temperatures were encountered about 2.5 mm away from the probe surface. These authors further demonstrated that beyond these hot spots, coagulation was mainly formed passively by thermal conduction. Lorentzen [35] had previously demonstrated that a concentric ring of char tissue at about 3 mm from the needle tract was often observed during ablation with internally cooled electrodes and a non-pulsed power algorithm (Fig. 8). Moreover, this charring tissue was linked to a significant fall in deposited power during ablation [9,35]. On the other hand, Goldberg et al [13] stated that saline perfusion may improve conductivity of the tissue and transfer heat by convection in hot spots during RFA. We therefore hypothesized that a hybrid applicator based on an internally cooled electrode combined with saline infusion into the tissue may be optimized in terms of deposited power and coagulation volume when the perfusion distance was precisely injected at these hot spots.

In our study we chose to assess three values of perfusion distance: 0, 2 and 4 mm. Results from the general linear model and best fit of least square functions confirmed that perfusion distance had a significant effect on both volume and minimum and maximum transverse diameters. In general, the best results were always obtained at a distance of 2 mm, showing at least a thirty per cent increase in volume of the coagulation necrosis over the other distances tested. Likewise, a significant increase of nearly twenty-five per cent was yielded in the 2 mm group over the 0 mm group in mean short diameter of the coagulation necrosis. This improvement could be important, taking into account that the short diameter may be one of the main determinants of technical success in RFA in a clinical setting [18]. In a similar experimental approach with ex vivo bovine livers, Lee et al [21] further demonstrated that the addition of infusion at 0-mm distance coupled with ICE may improve both the mean volume of coagulation necrosis, in comparison with a single ICE (from 13.1 to 43.7 cm^3^, respectively), and mean short diameter (from 2.6 to 3.4 cm, respectively). In our study we obtained similar figures in both the short diameter and volume for a perfusion distance of 0 mm (4.0 ± 0.4 cm and 53.0 ± 15.0 cm^3^, respectively) but these values were significantly improved when the perfusion distance was 2 mm (5.3 ± 0.5 cm and 91.7 ± 18.2 cm^3^, respectively). However, when the perfusion distance was increased, the short diameter of the lesion did not increase, in fact, the volume was significantly reduced. This phenomenon was also shown by the decreasing step of the hyperbolic function of volume size related to the perfusion distances > 2 mm (Fig. 5A). Concerning coagulation shape, infusion of saline at a distance from the ICE (2 mm and 4 mm groups), in contrast to around the ICE (0-mm group), seemed to increase sphericity of the lesion (mean sphericity ratio: 1.1 and 1.5, respectively). These figures match well with the results published by Pereira et al [10], who described a sphericity ratio of 1.38 for cluster cool-tip electrodes and 1.42 for RITA electrodes in a pig liver in vivo model.

The improvement in coagulation volume matched well with a significant increase in deposited power, lower relative frequency of uncontrolled impedance rise during ablation (Fig. 6) and lower relative frequency of char tissue in coagulation necrosis. It is possible that a more precisely focused saline infusion into the hottest area of ablation, with the double objective of improving conductivity and heat convection, could account for this improvement. Actually, diffusion of saline into the liver tissue has not yet been characterized, but according to the evidence from other tissues, may not be homogeneously guaranteed beyond 1 cm away from the injection site [36]. However, perfusion of saline at a single spot near the electrode may be beneficial, since the relative concentration of deposited power in the perfused spot may allow a reduction of deposited power at the non-perfused spots. In other words, perfusion of saline in a small area near the electrode may avoid overheating and sometimes charring of the rest of the tissue, even if this tissue is not reached by the saline itself.

### Limitations of the study

Several limitations inherent in the study should be pointed out:

1. This is an ex vivo study in healthy bovine livers. Therefore, the actual clinical setting may be substantially different, not only because of the known "heat sink effect" of blood perfusion but also because of tumor tissue characteristics.

2. Even though the pulsed power algorithm has been demonstrated to improve coagulation volume and deposited power [31], it probably reduces the risk of tissue charring. This algorithm was not employed in our study in order to facilitate impedance rises and recognition of charring. It is conceivable that the use of this algorithm could improve or modify certain results.

3. Precise perfusion of saline was performed by a perfusion needle held by means of two fixation systems attached to the electrode to guarantee the parallel position in the desired location. However, the exactitude of the distance between the ICE and infusion needle at the tip zone was not measured precisely.

4. The coagulation shape is of paramount importance in clinical practice and was evaluated by the sphericity ratio in this work. Although a more comprehensive evaluation of the coagulation shape should be mandatory in upcoming research, it is outside the scope of this article.

### Practical application

While using internally cooled electrodes for RFA, coupled with perfusion of saline into the tissue, the proper location of the saline perfusion, as discussed in this study, should enhance and focus energy dissipation in the targeted tissue. This configuration should lead to better performance in liver tumor RFA.

## Conclusion

Perfusion of saline at around 2 mm from the electrode surface while using an internally cooled electrode in RFA improves deposition of energy and coagulation volume.

## Competing interests

Drs. F. Burdío, E.J. Berjano, A. Navarro and A. Güemes are applying for a patent relating to the content of the manuscript. All other authors declare that they have no competing interests.

## Authors' contributions

FB conceived the study and participated in its design and coordination; EJB and JMB participated in the design of the study and performed the statistical analysis; AN participated in the design of the study and carried out the experiments; AGU, RS, JS, AGO, IC and ET carried out the experiments; LG participated in the design of the study and helped to draft the manuscript; TC conducted the histopathologic examinations; RL and MG participated in the design of the study. All authors have read and approved the final manuscript.
